# Disorders of the Aorta and Aortic Valve in Connective Tissue Diseases

**DOI:** 10.1007/s11886-020-01314-0

**Published:** 2020-06-19

**Authors:** Bogna Grygiel-Górniak, Mary-Tiffany Oduah, Abdulbaril Olagunju, Michal Klokner

**Affiliations:** Department of Rheumatology and Internal Diseases, Poznan University of Medical Sciences, Poznan, Poland

**Keywords:** Aortic valve diseases, Connective tissue diseases, Diagnosis, Treatment

## Abstract

**Purpose of Review:**

The incidence of aortic valve disease in inherited connective tissue disorders is well documented; however, recent studies have only begun to unravel the pathology behind this association. In this review, we aim to describe the etiology, clinical manifestations, management, and prognosis of aortic and aortic valvular disorders that co-exist in a variety of connective tissue diseases. An extensive literature review was performed in PubMed. Articles from 2008 to 2018 were included for review. Predetermined search terms used in PubMed include “aortic manifestation of connective tissue diseases” and “aortic valve disorders in rheumatologic disease.”

**Recent Findings:**

Manifestations of aortic valve disease in the context of connective tissue disorders include valvular stenosis, regurgitation, and/or thoracic aortic aneurysms. Both inherited and inflammatory connective tissue disorders contribute to aortic valve damage with increased susceptibility associated with specific gene variants.

**Summary:**

Anti-inflammatory and immunosuppressive therapies have demonstrated beneficial results in Marfan’s syndrome, Behcet disease, rheumatoid arthritis, ankylosing spondylitis, and systemic sclerosis, often leading to remission. Yet, such therapy is less effective in other disorders compared to alternative treatments such as surgical intervention. Additionally, regular echocardiographic studies should be recommended to those suffering from these disorders, especially those at higher risk for cardiovascular involvement.

Given the rates of relapse with immunosuppressants, even following aortic valve replacement, further studies are needed to determine if certain dosing and/or combinations of immunosuppressants could be given to those diagnosed with connective tissue diseases to prevent progression of aortic valve involvement.

## Introduction

Aortic and aortic valve disorders manifesting as aortic valve sclerosis, stenosis, insufficiency, bicuspid aortic valve, aortitis, and aortic aneurysms occur in the setting of rheumatic diseases. There is a paucity of recent studies, which seek to unravel the underlying mechanisms for increased cardiovascular disease risk in rheumatologic diseases. In this review, we aim to describe the etiology, clinical manifestations, management, and prognosis of aortic valvular disorders that are present in a variety of connective tissue diseases.

Manifestations of aortic valve disease in the context of connective tissue disorders include valvular stenosis, regurgitation, and/or thoracic aortic aneurysms (Tables 1 and 2). Both inherited and inflammatory connective tissue disorders contribute to aortic valve damage with increased susceptibility associated with specific gene variants. There is insufficient knowledge regarding the underlying mediators, and thus appropriate immunotherapy to target specific cytokines in order to dampen the inflammatory response. For example, in Marfan’s syndrome, TGF-β has been documented to play a crucial role and thus presents a therapeutic target. Ultimately, the goal of such therapies would be the induction of remission without the need for valve replacement, which is associated with inherent risk.

**Table 1:** Connective tissue diseases and their manifestations in the aorta and aortic valve

Aortic disorders and aortic valve manifestations	Connective tissue diseases
Aortic valve regurgitation	• Ankylosing spondylitis • Churg–Strauss syndrome • Behcet disease • Ehlers–Danlos syndrome • Takayasu vasculitis • Primary antiphospholipid syndrome
Aortic valve stenosis	• Systemic sclerosis • Primary antiphospholipid syndrome-related bioprosthetic valve stenosis
Aortic valve stenosis and regurgitation	• SLE • Sjogren syndrome
Aortic valve thickening	• Rheumatoid arthritis • Granulomatosis with polyangiitis
Dilation of aortic root and/or ascending aorta; dissecting thoracic aortic aneurysms; aortic valve regurgitation	• Marfan’s syndrome

**Table 2:** Aortic valve changes in certain vasculitis

Name of vasculitis	Takayasu arteritis	Granulomatosis with polyangiitis	Churg–Strauss syndrome	Behcet disease
Type of vasculitis	Large vessel arteritis	ANCA-associated small vessel vasculitis	ANCA-associated small vessel vasculitis	Various vessel vasculitis
Aortic involvement	Aortic regurgitation	Aortic regurgitation (rarely aortic stenosis)	Mainly aortic regurgitation	Aortic regurgitation Aneurysms Pseudoaneurysms Aortic rupture Stenotic lesions (brachiocephalic artery)
Characteristic of aortic valve changes	• Secondary to dilation of the aortic root • Normal valvular cusps in echocardiography shows • Aneurysm can be present	• Thickened cusps of the aortic valve • May be the only sign of relapse in GPA • The onset: at the time of diagnosis or few years after diagnosis and correlates with elevated PR3-ANCA • Chronic or acute manifestations (e.g., the perforation of the cusps of the aortic valve)	The valve is dense and thick due to fibrosis and the inflammatory reaction	Aortic regurgitation • The most common valvular pathology • Aneurysm • Elongation and prolapse of aortic cusps • Vegetation like lesions Aortic valve regurgitation • Can be secondary to aneurysm of the ascending aorta due to vasculitis • Normal or fibrosed cusps of the valves • Annulus dilatation • Cusp fenestration and fusion
Histopathologic findings	• Disrupted elastic fibers in the media • Granuloma • Marked collagen deposition in the adventitial	• Granulomatous inflammation • Polymorphonuclear microabscesses (differentiate with infective endocarditis) • Foci of necrosis • All findings located in the central layer of the valve	• Fibrosis and inflammation => dense and thick valve • Valve leaflets infiltrated by eosinophils, lymphocytes and plasma cells • Necrotizing granulomas	Lymphoplasmacytic infiltrates, neutrophils, histiocytes, eosinophils and occasional giant cells, myxoid degeneration, focal necrosis, and fibrotic thickening
Treatment	• Aortic valve replacement • Aortic root replacement	• Immunosuppressive therapy used in GPA - No impact on the development of aortic regurgitation • Increasing dose of cyclophosphamide => Slows down the progression of aortic regurgitation	Improvement after • Prednisolone • Cyclophosphamide • Aortic valve replacement if relapse is present	• Aortic valve replacement • Pharmacologic treatment: anticoagulants, immunosuppressive therapy, colchicine

Despite demonstrating promising results in the treatment of Ehlers–Danlos syndrome (EDS), surgical intervention of the aortic valve has been associated with many complications, including the need for recurring operations in Behcet disease and ankylosing spondylitis and a high mortality and morbidity rate in systemic lupus erythematosus (SLE).

Given that patients experience relapse when placed on immunosuppressants, even following aortic valve replacement, further studies are needed to determine if certain dosing regimens and/or combinations of immunosuppressants are helpful in preventing progression to aortic valve involvement.

## Aortic Valve Manifestations in Rheumatic Diseases

### Rheumatoid Arthritis

**Fig. 1:**
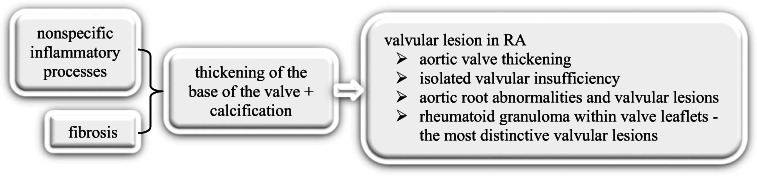
Overview of aortic valve disorders in rheumatoid arthritis

Rheumatoid arthritis (RA) is a chronic, autoimmune, inflammatory disorder that primarily affects synovial joints, with a prevalence of 0.53–0.55% in the USA [1••, 2]. The disease is progressive, eroding cartilage and bone, resulting in joint deformities, severe disability, and ultimately early mortality. Although the central pathology of RA pertains to the synovium of diarthrodial joints, many non-articular organs are affected in chronic cases, including the heart. [3] One systematic review reported a 48% increased risk for CVD in patients diagnosed with RA, a 60% increased risk of CV death, and a reduced life expectancy of 3 to 10 years, compared to the general population. [4] Additionally, the pattern of CVD in RA patients differs from the general population, as they are more likely to develop silent ischemic heart disease, develop heart failure with increased morbidity and mortality [5]. Additional markers of vascular disease in RA include aortic valve thickening, isolated valvular insufficiency, aortic root abnormalities, and other valvular lesions. [6] Fig. 1 illustrates underlying mechanisms leading to valvular lesions in RA.

Moreover, the most distinctive valvular lesions are rheumatoid granuloma within valve leaflets, occurring as a result of nonspecific inflammatory and fibrotic reactions at the base of the valve that causes thickening and calcification [7]. Furthermore, when inflammation cannot be adequately controlled in such conditions, fibrosis is observed in the valvular base, followed by calcification and finally valvular lesion. [8] Given that the suppression of RA-associated inflammation has been shown to not only decrease lipid levels but also reduce CV events, anti-inflammatory medication has demonstrated beneficial results as a treatment method [9]. One study found that the use of anakinra, an IL-1 blocker, improved CV function in RA patients as early as 3 h after administration of the first dose, a result that was sustained after 30 days of regular use [10•]. Furthermore, anti-TNF biologics have become fairly standard for treating the CV manifestations of RA. Vizzardi et al. reported that a 1-year treatment with anti-TNF-α drugs significantly reduced systemic inflammation, aortic wall stiffness, and the overall activity of the disease [11].Future studies are needed to better understand the effect of treatment on the course of cardiovascular complications related to rheumatoid arthritis.

### Systemic Lupus Erythematosus and Antiphospholipid Syndrome

Systemic lupus erythematosus (SLE) is a chronic inflammatory disease, which affects all organ systems [12•]. It is classified as a systemic autoimmune disease of unknown etiology, with a prevalence of 20–70 per 100,000 that is 6 times greater in women than men, and a peak onset between 15 and 40 years of age [13–15]. SLE is characterized by over-reactive and auto-reactive T cells, which distort normal cytokine production, resulting in increased inflammation and multi-organ tissue damage. Furthermore, B cells are also over-reactive, producing excessive amounts of autoantibodies, which form immune complexes that ultimately deposit into various organs and disrupt normal tissues functions [16]. The heart is an example of such an organ, given the high prevalence of cardiovascular disease in SLE of over 50%. [17]

Valvular abnormalities are quite prevalent in SLE patients, including aortic valve regurgitation, which was reported to be one of the most common valvular impairments observed. [18] One of the most prevalent cardiac manifestations of SLE is Libman–Sacks endocarditis with 1–4 mm large, verrucous vegetations present on either side of the aortic and mitral valves [19].

**Fig. 2:**
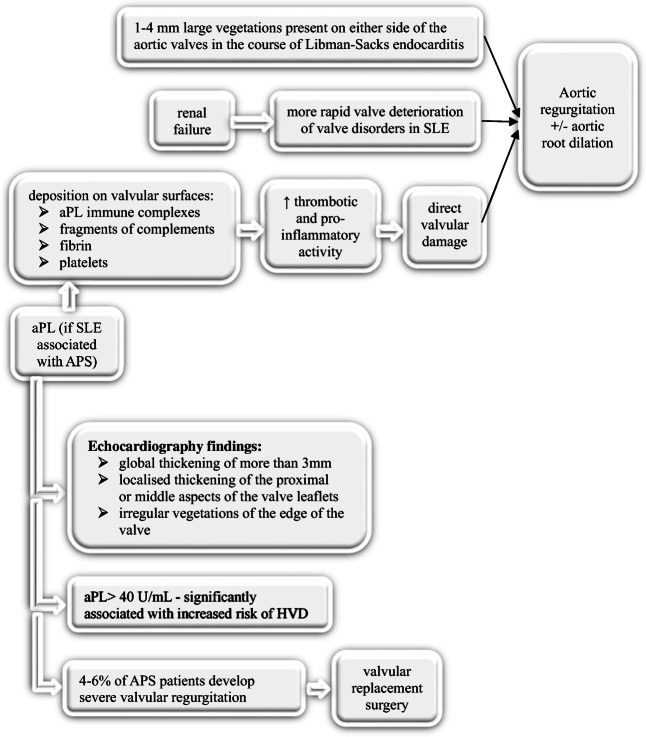
Overview of aortic valve disorders in antiphospholipid antibody syndrome

Elevated levels of antiphospholipid antibodies (aPL) are present among SLE patients with antiphospholipid syndrome (APS) often presenting as an arterial or venous thrombosis. Multiple studies have discovered aPL immune complexes, fragments of complements, and fibrin and platelets within valvular vegetations in SLE patients presenting with Libman–Sacks endocarditis [19]. This is thought to occur as a result of aPL immune complexes depositing on valvular surfaces, ultimately leading to direct valvular damage through thrombotic or inflammatory mechanisms (Fig. 2). Moreover, one study found that increased levels of aPL antibodies, specifically higher than 40 U/mL, were significantly associated with increased risk of heart valve disease (HVD), further supporting this theory [19]. Additionally, it was observed that valve lesions in Libman–Sacks endocarditis patients were more severe when accompanied by APS and that patients with APS required surgical intervention more frequently, with a greater perioperative risk. [20]

Several treatments for SLE are available, yet there is currently no cure [21]. One study recommends regular screening via echocardiogram for patients with elevated aPL levels, as they appear to be more at risk. In this study, the use of echocardiogram is demonstrated to be an effective diagnostic tool for valvular abnormalities in SLE, as it uncovered valvular defects in 61% of SLE patients. Moreover, the authors noted that trans-esophageal echocardiograms are capable of generating higher resolution images of valvular damage as compared to trans-thoracic echocardiograms and thus are preferable in such diagnosis [19]. Transesophageal echocardiography (TEE) is recommended if degree of valve pathology is not clear on transthoracic echocardiography or for surgical planning. Additionally, aggressive anticoagulant or anti-platelet therapy is recommended by some experts, due to the presence of valvular vegetations in SLE, which may augment the development of thromboembolic complications, such as stroke [20].

A recent study has recommended the use of cardiovascular magnetic resonance (CMR) as the first line, non-invasive cardiac imaging modality, due to its ability to detect cardiovascular involvement in 43.3% of SLE patients [12•]. This low rate of false negatives associated with CMR is a result of its capability in detecting subclinical cardiac involvement, as only 20% of asymptomatic patients exhibit left ventricular abnormalities [12•]. Given the often asymptomatic nature of valve abnormalities in SLE patients, it was reported that only 4–6% of APS diagnosed patients develop severe valvular regurgitation that requires valvular replacement surgery. However, this rate rises when renal failure is involved, as it causes more rapid valve deterioration. [17] Moreover, one retrospective analysis concluded that cardiac surgery in SLE confers a high rate of mortality and morbidity, with early and late death reported in 17% and 10% of patients, respectively. The main cause of these deaths was sepsis, with at least one postoperative complication occurring in 63% of cases. The authors thus recommend that preoperative risk scores specific to SLE be developed to help guide medical and/or surgical management. [22]

### Systemic Sclerosis

Systemic sclerosis is an autoimmune disease with unknown triggers and is characterized by aberrant interstitial and perivascular fibrosis of multiple organs: the skin, GI, kidneys, lungs, and heart muscle [23]. The heart valves are less commonly affected with mitral valve prolapse and aortic stenosis most commonly encountered in scleroderma [24]; the latter being the rarer.

Within the last 10 years, 3 cases of systemic sclerosis with aortic stenosis have been described in the literature. The first 2 cases were of women aged 59 and 73 that had worsening dyspnea due to aortic stenosis demonstrated by echocardiography. The younger patient had limited scleroderma with autoimmune liver cirrhosis complicated by esophageal varices, while the older patient had systemic sclerosis, Sjogren syndrome, mild pulmonary hypertension, antiphospholipid antibody syndrome, and multi-nodular goiter [24]. The third case was that of a 69-year-old woman that presented with severe dyspnea due to aortic stenosis diagnosed via trans-thoracic echocardiography; she had a trans-aortic gradient of 45 mmHg and valve area of 0.9 cm^2^. She had limited scleroderma without lung involvement demonstrated by CT lung imaging [25]. The aortic valves of the 3 patients had 3 different histopathologic findings. Examination of the aortic valve from the first patient revealed only dense calcification, while that of the second patient revealed calcification along with fibrin and platelet deposits. The histologic examination of the third patient’s aortic valve had dense, acellular fibrosis with few calcifications. Based on these findings we may assume that the pathogenesis of aortic stenosis in scleroderma could take the form of aberrant fibrosis similar to other organs in the body. As with the third patient, it could be as a result of chronic hemodynamic stress on the valve due to elevated afterload in scleroderma, and this is likely suggested by the dense calcification of the valve of the first patient, which is also responsible for aortic stenosis in senile aortic stenosis. Valvular fibrosis may also be due to comorbidities presenting with scleroderma, as the fibrin and platelet deposits in the second patient may be secondary to antiphospholipid antibody syndrome [24].

Hence, aortic stenosis in scleroderma does not have a specific pathogenesis. The three patients were managed by replacing their aortic valves with biological prostheses [24, 25] The first and second patients were asymptomatic after the surgery and were discharged after 7 days, and upon follow-up after 38 months and 12 months, they were both stable. No infectious complications were noted in both patients after the surgery despite being on corticosteroids. They were also placed on ACE inhibitors after the surgery because they slowed down collagen synthesis [24]. The recovery of the third patient was also uneventful and was discharged on day 6 after the operation. The patient was not on corticosteroid and did not have infectious complications. Follow-up after 1 year was positive; the patient was in sinus rhythm and in NYHA class 1 and well-functioning prosthesis with a mean trans-prosthetic gradient of 15 mmHg [25].

### Sjogren Syndrome

Sjogren syndrome is characterized by chronic lymphocytic infiltration of salivary glands, lacrimal glands, and other exocrine glands in the body that results in dryness, burning, and pruritus of the eyes and blurriness of vision. Patients also suffer from xerostomia and its consequences such as dysphagia, decreased taste sensation, and cracks and fissures in the mouth. SS is highly associated with autoantibodies SS-A and SS-B [23].

Although the aortic valve is rarely affected in SS, aortic stenosis with or without regurgitation has been described as the echocardiographic findings in SS with aortic valve involvement [26]. Patients present with the main complaints of exertional dyspnea, syncope, and chest pain. The aortic valves appear stiffened, calcified, and have restricted motion on echocardiography [26, 27].

The cases of SS with aortic involvement described in the literature are above age 60, and the echocardiographic and gross examination findings could also be attributed to aortic valve sclerosis, which is the manifestation of atherosclerosis at the level of the aortic valve, which is associated with aging. However, histopathologic examination may help exclude aortic valve sclerosis as the primary cause of the patients’ aortic valve disease as lipid-laden macrophages present in aortic valve sclerosis [28] would not be present if SS was the sole cause of the stenotic features.

If SS is the primary cause, nodular calcifications and hyaline degeneration with myxomatous degeneration with or without lymphocytic infiltration would be the predominant histopathologic findings. The absence of lymphocytic infiltration has been attributed to glucocorticoid use. However, in most cases, the primary cause of stenosis is not definitive because patients might have a history of rheumatic heart disease or aortic valve sclerosis in addition to SS [26]. Anti-inflammatory medications such as glucocorticoids and hydroxychloroquine were associated with the improvement of symptoms as well as echocardiographic findings in a patient with SS [27].

## Aortic Valve Disorders in Seronegative Spondyloarthropathies

### Ankylosing Spondylitis

Ankylosing spondylitis is a disease characterized by sacroiliitis, enthesitis of the vertebral bodies (lumbar) that results in their fusion. It is more common in men with an onset in the second and third decades; lower back pain and stiffness that improve with exercise are typical complaints. About 90% of those affected are HLA-B27 positive [23]. Aortic valve diseases in ankylosing spondylitis are aortic insufficiencyregurgitation and rarely stenosis and regurgitation of bicuspid aortic valve [29–31]. Overall, 80% of patients with ankylosing spondylitis have aortic valve and/or aortic root involvement [32].

**Fig. 3:**
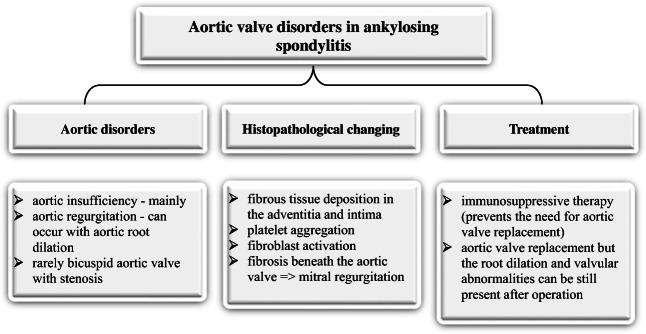
Overview of aortic and aortic valve disorders in ankylosing spondylitis

Aortic regurgitation can occur with aortic root dilation; for example, a 44-year-old male patient who was found to have aortic root dilation of 5 cm and severe regurgitation on echocardiography; the patient was managed by valve replacement. Histopathological examination of the aortic valve cusps, root, and sinuses revealed fibrous tissue deposition in the adventitia and intima. Platelet aggregation in the inflammatory site is thought to be involved in fibroblast activation. Fibrosis was also present beneath the aortic valve and is responsible for the subaortic ridge, which can be detected on echocardiography. The subaortic ridge could extend to the anterior leaflet of the mitral valve and may prevent closure of the leaflet resulting in mitral regurgitation [29]. Figure 3 summarizes aortic lesions seen in ankylosing spondylitis.

Aortic valve replacement may be a way to manage patients with ankylosing spondylitis who present with signs and symptoms of heart failure secondary to aortic regurgitation. However, autopsy findings in 5 of 8 patients who underwent aortic valve replacement still revealed root dilation and valvular abnormalities consistent with aortic regurgitation [29]. Although the nature of the valves used was not stated, such findings indicate the need for recurrent valvular replacement in those with seronegative spondyloarthropathy such as ankylosing spondylitis or psoriatic arthritis [31, 33]. Interestingly, a study of a patient with ankylosing spondylitis revealed that immunosuppressive therapy prevented the need for aortic valve replacement in addition to slowing the progression of joint inflammation [31]. Only a single case of bicuspid aortic valve stenosis in ankylosing spondylitis has been described in the literature in the last 10 years. The patient was a 65-year-old male with aortic dilation of 5 cm and stenosed bicuspid aortic valve with calcification detected via echocardiography. However, it was not stated if the bicuspid valve was congenital and developed chronic calcification as a result of stress or if the patient had a tricuspid valve that degenerated to take the form of bicuspid valve on echocardiography as a result of ankylosing spondylitis. There are no specific recommendations for ankylosing spondylitis; however, it is reasonable to follow diagnostic and treatment options used in other diseases known to cause aortic regurgitation and/or root dilation [32].

### Reactive Arthritis

Reactive arthritis (ReA) is an immune-mediated oligoarthritis that develops secondary to a gastrointestinal or genitourinary tract infection. Urethritis and conjunctivitis are also common in those with ReA. Although the pathogenesis of ReA is not well understood, it is believed to be an interplay between the presence of bacterial antigens in the joints, the interaction between the host and bacterial antigen and the immune response against the antigens. It has also been thought that some of the bacterial antigens are molecularly similar to some self-antigens present in other body parts affected in ReA and hence this molecular mimicry results in the immune cells targeting the host tissues. ReA is strongly associated with HLA-B27 genotype; this has been partly explained by the fact that HLA-B27 shares some amino acid sequences with the bacteria that GI/GU infections [34]. However, ReA is also sometimes seen in patients that lack the HLA-B27 phenotype [35]; however, the underlying mechanism is yet to be determined.

The valvular manifestation in ReA is aortic regurgitation. The time from the diagnosis of ReA to development of aortic regurgitation varies among patients. On average, it is usually preceded by musculoskeletal involvement by 13 years; however, it may be detected from 4 days to 61 years after diagnosis. Patients may be asymptomatic and be detected during routine physical. ECG findings may include left ventricular hypertrophy and left axis deviation/fascicular block. Findings on echocardiogram may include trileaflet aortic valves that move as bileaflet due to commissural fusion, aortic root dilation, left ventricular dilation with hypertrophy, and left ventricular systolic dysfunction [36]. In a case that was negative for HLA-B27, diffuse aortitis and annular ectasia were discovered during operation; valvular biopsy revealed myxomatous degeneration without inflammatory infiltrates [35]. Once detected, routine follow-up is necessary to monitor the progression and assessment for aortic valve replacement indications. Some patients may be asymptomatic; however, the extent of aortic valve deterioration between consecutive follow up in some has warranted the need for replacement despite being asymptomatic [36].

Little is known regarding the prognosis of patients with ReA after aortic valve replacement. In 2 cases, repeated echocardiography 4 and 5 years following aortic valve replacement demonstrated normal valvular function without relapse. However, a case was reported with severe calcification of a bovine bioprosthetic aortic valve after 7 years of replacement. The patient was also found to have severe coronary calcification during the procedure and required CABG. It was thought that the underlying degree of inflammation was responsible for the continued calcification [36]. Therefore, more studies are needed in which patients are followed for a longer period to determine if they suffer a relapse and to determine the factors that contribute to the severity of aortic involvement.

## Aortic Valve Disorders in Vasculitis

### Takayasu Arteritis

Takayasu arteritis is a vasculitis of unknown etiology that predominantly affects the aorta, the great vessels, and the pulmonary arteries [23]. There is a chronic inflammation of the arterial wall, which is transmural and characterized histologically by mononuclear cell infiltrates and fibrosis of the vessel wall that results in stenosis of the lumen in some cases and aneurysm of vessel wall in a few cases [23]. Aortic regurgitation is the valvular disorder that occurs in Takayasu arteritis [37, 38] and is the result of aortic root dilation. Echocardiogram shows normal valvular cusps [37]. Management is aortic valve replacement; however, aneurysm of the right sinus of Valsalva (discovered incidentally via echocardiogram during routine follow-up) developed 14 years after valve replacement in a patient despite being on daily prednisone. Histopathologic examination of the sinus revealed disrupted elastic fibers in the media, granuloma, and marked collagen deposition in the adventitial. The patient underwent aortic root replacement [38].

The 2010 AHA guidelines recommend thoracic aorta imaging with CT or MR for detection of aneurysms or luminal stenosis [32]. Imaging of the entire aorta with CT or MR angiography is recommended in suspected cases of aortic aneurysm [39].

### Granulomatosis with Polyangiitis

This is a proteinase 3 anti-neutrophilic cytoplasmic antibody (PR3-ANCA) positive necrotizing vasculitis characterized by the presence of necrotizing granuloma of the upper and/or lower respiratory tract, necrotizing or granulomatous vasculitis of small- and medium-sized vessels in lungs, upper airways, and other organs and crescentic glomerulonephritis [40]. Although valvular involvement in the disease is quite rare, aortic regurgitation and aortic stenosis have been reported in patients with granulomatosis with polyangiitis (GPA) with aortic regurgitation being more prevalent [41, 42].

The onset of aortic regurgitation in GPA is variable because in some cases, it was present at the time of diagnosis, and in other cases, it presented a few years after diagnosis. More so, the immunosuppressive therapy used in GPA seems to have no impact on the development of aortic regurgitation because some patients may develop symptoms of aortic regurgitation despite being on immunosuppressive therapy to control the disease progression [41]. Both chronic and acute manifestations could be present (e.g., the perforation of the cusps of the aortic valve) [42]. Aortic regurgitation may be the only sign of relapse in GPA patients that were in remission due to immunosuppressive therapy. Furthermore, the onset of aortic valve regurgitation correlates with elevated PR3-ANCA [43].

Macroscopically, affected aortic valves have thickened cusps. On histopathologic examination, dominant findings are granulomatous inflammation with polymorphonuclear micro-abscesses and foci of necrosis [44]. These findings are located in the central layer of the valve. Echocardiography, blood and valve cultures, and serology should be done in patients with GPA with valvular involvement to rule out infectious endocarditis. The absence of vegetations, negative blood and valve cultures, and serology and the absence of microbes on histopathologic examination were documented in one study of patients with aortic regurgitation secondary to GPA [45].

Cases of aortic regurgitation in GPA have been managed with either increasing the dose of immunosuppressive therapy or aortic valve replacement. Increasing the dose of cyclophosphamide did slow down the progression of aortic regurgitation in a few cases. However, increasing the dose of rituximab did not improve the findings in a few patients with valvular granulomas detected via transesophageal echocardiography. Rather aortic valve replacement was performed on these patients [46] The differences in the potency of cyclophosphamide and rituximab or severity of valve involvement may be the reason why not all cases respond to an increased dose of immunosuppressive therapy. Perhaps more studies need to be carried out on the most effective immunosuppressive therapy for those with GPA and aortic regurgitation. Follow-up studies need to be done on these patients to determine the effectiveness of valve replacement, i.e., if the disease recurs or not due chronicity of GPA. Without randomized studies, it is difficult to broadly apply these reports of outcomes from medical treatment or surgical valve replacement to patient care.

### Churg–Strauss Syndrome

Churg–Strauss syndrome (CSS) is a systemic necrotizing granulomatous vasculitis of small to medium muscular arteries and microvessels and is associated with asthma, allergic rhinitis, peripheral eosinophilia, and lung infiltrates. Myeloperoxidase antineutrophil cytoplasmic antibody (MPO-ANCA) is present in less than 50% of cases [23]. The aortic valve pathology associated with CSS is aortic regurgitation [47, 48]. Histopathologic examination reveals valve leaflets infiltrated by eosinophils, lymphocytes and plasma cells; necrotizing granulomas are also seen. Grossly, the valve is dense and thick due to fibrosis and inflammatory reaction [48]. Anti-inflammatory medications like prednisolone and cyclophosphamide resulted in clinical improvement in a patient with aortic regurgitation secondary to CSS. However, the patient later required aortic valve replacement due to relapse [47]. Further studies are needed to understand the role of medical therapy on the impact of development and/or progression of valve disease associated with CSS.

### Behçet’s Disease

Behçet’s disease is a chronic vasculitis involving multiple organs and is diagnosed based on the presence of recurrent oral aphthous ulcers and at least 2 of skin manifestations, genital ulcerations, eye lesions, and a positive pathergy test [49]. It is associated with HLA-B51 [50]. About 25% of patients with Behçet’s disease have valvular involvement, and aortic regurgitation is the most common valvular pathology [51, 52]. The echocardiographic findings are aneurysm, elongation and prolapse of aortic cusps, echocardiographic free space, and vegetation like lesions [53, 54]. These findings are not unique to Behçet’s disease, however, and may be seen in different forms of endocarditis, which have to be excluded.

Although the exact pathology and molecular basis of aortic valve regurgitation in Behcet disease is unknown, regurgitation may be secondary to aneurysm of the ascending aorta due to vasculitis [51, 52]; the cusps of the valves maybe normal or fibrosed. Grossly visible changes include annulus dilatation, sinus of Valsalva aneurysm, cusp fenestration, and fusion. Histopathologic evaluation of the valve and ascending aorta may reveal lymphoplasmacytic infiltrates, neutrophils, histiocytes, eosinophils and occasional giant cells, myxoid degeneration, focal necrosis, and fibrotic thickening [52, 54].

Some patients will meet indications for aortic valve replacement due to severe aortic valve regurgitation, and some may need concomitant aortic root or ascending aortic replacement. The role of immunosuppressive therapy to prevent valve degeneration is the focus of ongoing research [55]. There are no specific recommendations for Behcet disease; however, it is reasonable to follow diagnostic and treatment options used in other diseases known to cause aortic regurgitation. Special focus should be on immunosuppressant therapy, which helps lessen the inflammatory state.

## Aortic Valve Disorders in Heritable Connective Tissue Diseases

### Marfan’s Syndrome

**Fig. 4:**
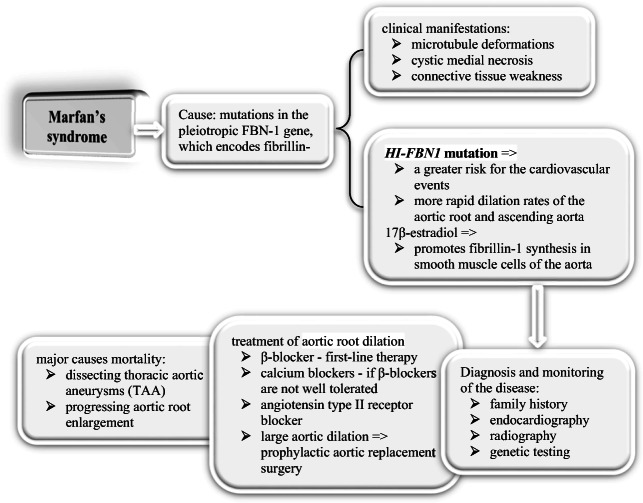
Overview of aortic valve disorder in Marfan’s syndrome

Marfan’s syndrome (MFS) is an autosomal dominant connective tissue disorder with cardiovascular involvement [56, 57]. With a prevalence of around 1/3000 to 1/5000 individuals, the cause of the disorder has been extensively linked to mutations in the pleiotropic FBN-1 gene, which encodes an extracellular matrix protein (ECM), fibrillin-1 [58]. Fibrillin-1 is critical for microfibril formation and organizing ECM components, as well as sequestering transforming growth factor (TGF)-β, thus regulating its bioavailability [59]. The induction of TGF-β signaling, through increased angiotensin II receptor signaling as a result of fibrillin-1 underproduction, is associated with an increase in apoptosis and subsequent loss of smooth muscle nuclei [57]. Moreover, antagonism of TGF-β by antibodies has exhibited some improvement with regard to the pulmonary manifestations of the disease [60]. Thus, the increased activity of TGF-β observed in MFS is believed to play a pivotal role in the adverse clinical manifestations of the disorder, which include microtubule deformations, cystic medial necrosis (CMN), and overall connective tissue weakness [61]. Figure 4 summarizes the mechanisms leading to aortic lesions seen in Marfan’s syndrome.

MFS demonstrates clinical variability, as phenotypes range from very mild to severe, even among family members with FBN-1 gene mutations [62]. It is suggested that deletions in various regions of the gene often result in different clinical phenotypes [58]. Franken et al. demonstrated that patients with a haploinsufficiency (HI) mutation in the *FBN1* gene were found to have more rapid dilation rates of the aortic root and ascending aorta, in contrast to dominant negative (DN) form of the mutation. About 79% of patients with cardiovascular events were HI-*FBN1* mutants, whereas only 48% of patients without these events belonged to this mutant type. This demonstrates that patients possessing the *HI-FBN1* mutation are at a greater risk for the combined clinical endpoint, compared to those with the *DN-FBN1* mutation, a finding consistent even at younger ages [63].

Cardiovascular abnormalities, such as dissecting thoracic aortic aneurysms (TAA) and progressing aortic root enlargement, are major causes of morbidity and mortality seen in MFS. These cardiovascular deficits may present either in neonatal life, where they are often fatal, or in adolescence, and worsen with age [64] Furthermore, the impact of gender and pregnancy on the cardiovascular implications of MFS has been increasingly studied [65].

One study found that men below the age of 30 were at a greater risk than women for aortic dilation and aortic events [66], while another study discovered an increased incidence for aortic surgery at baseline in men (38%), compared to women (19.4%) [67]. The mechanism to explain the influence of gender on cardiovascular disease in MFS was subsequently investigated by Renard et al. who showed that 17β-estradiol promoted fibrillin-1 synthesis in smooth muscle cells of the human aorta [65]. Due to improved medical and surgical therapies for aortic dilation, the life expectancy of those suffering from MFS has increased from 47 to 75 years. Echocardiography should be performed at the time of diagnosis. Definitive diagnosis as well as surveillance imaging is accomplished with CT or magnetic resonance imaging of the entire aorta [32, 39]. CT angiography or magnetic resonance angiography (CTA or MRA) of the entire aorta is needed for diagnostic imaging [39]. Furthermore, genetic testing has evolved into an essential diagnostic tool of the disease, owing to its 97% effectiveness in its ability to detect *FBN1* mutations. Cumulatively, genetic testing, radiography, and family history are currently utilized in diagnosing MFS [68]. For patients with familial thoracic aortic aneurysms, screening of first degree relatives (every 5 years) is prudent to prevent premature death [32, 39].

MFS patients with severe progression of the disease, resulting in aortic dilation, may undergo prophylactic aortic replacement surgery, which replaces the diseased aorta with a Dacron graft. Surgical intervention is recommended when aortic aneurysm reaches 4.5 to 5.0 cm in diameter [39, 69]. Additionally, current guidelines recommend endocardiography for children and those with accelerated aortic root growth twice a year. In order to manage the cardiovascular manifestations of the disease, β-blocker medication is generally used as the first-line of therapy, while calcium blockers are prescribed to those patients who cannot tolerate β-blockers [64]. However, several other potential therapeutic drugs are currently being investigated, including angiotensin type II receptor blockade, which minimizes TGF-β activity. Initial results are promising, with one study citing no difference between β-blocker use and angiotensin type II receptor blockade on aortic root dilation [70]. According to the 2010 AHA and 2014 ESC guidelines, prophylactic use of beta-blockers, angiotensin II receptor blockers, and angiotensin converting enzyme inhibitors is recommended to control blood pressure. This slows the progression of aortic dilation as well improves survival in this population [32, 39].

### Ehlers–Danlos Syndrome

Manifestations of EDS stem from the genetic defect in the synthesis and processing of collagen, which affects the tensile strength and integrity of connective tissues [23]. Based on clinical features, there are six types of EDS, which are the classic (with skin involvement such as easy bruising and atrophic scars and hypermobility of joints), hypermobility (hypermobility, pain and dislocation of joints), vascular (involving arterial and hollow organ rupture, thin skin that bruises easily, and hyperextensibility of small joints), kyphoscoliosis (congenital scoliosis, hypotonia, joint hypermobility, and ocular fragility), arthrochalasia (severe laxity of joint, mild skin involvement with bruising, and scoliosis), and dermatosparaxis EDS (comprising of severe skin involvement with cutis laxa and bruising) [71].

Aortic regurgitation is the aortic valve disorder present in EDS and has been associated with the classic, vascular, kyphoscoliosis, and arthrochalasia EDS [72–74]. Apart from mild asymptomatic aortic regurgitation, which is discovered during echocardiography for symptomatic mitral valve regurgitation in patients with EDS, patients present with exertional dyspnea, orthopnea, and palpitations [74].

Aortic regurgitation in EDS mostly appears to be secondary to aortic root dilation [72, 74, 75]. Patients with mild aortic regurgitation are monitored regularly because aortic regurgitation in EDS is often progressive [73]. Patients with symptomatic severe aortic regurgitation are offered surgical replacement [75]. The guidelines recommend surgical intervention when aortic size is 4–5 cm in diameter [32]. ESC guidelines have no set cutoffs, however, recommend lower threshold than 5.5 cm for surgical intervention [39].

Type 4 EDS (also known as vascular EDS) occurs due to mutations in type 3 collagen (COL3A1 gene). It is strongly associated with increased aortic friability and fragility leading to vascular complications such as aortic dissections and rupture, at a very young age [76]. Consequently, non-invasive screening modalities (CTA or MRA) are preferred over invasive angiography in this population [32, 77]. Surgical intervention is recommended at aortic diameter 4.4 cm [77]. In addition, the guidelines recommend excision of the sinuses in combination with a modified David reimplantation operation in these patients with dilatation of the aortic root and sinuses of Valsalva where possible [32].

### Loeys–Dietz Syndrome

Loeys–Dietz syndrome (LDS) is a autosomal-dominant disorder that occurs as a result of loss-of-function mutations of genes involved in the TGF-β signaling pathway that ultimately lead to increased TGF-β signaling [72]. It is characterized by craniofacial abnormalities, arterial tortuosity, and aortic root aneurysm [72, 78]. Thoracic aortic aneurysms (TAA) in LDS are prevalent at narrower aortic diameters, including medium-sized arteries [79]. In addition to arising at a smaller diameter, these aortic ruptures often occur at an earlier age, making LDS clinically distinct from MFS, with a mean life expectancy of 26 years [78]. Moreover, for patients with LDS, pregnancy is considered a high risk, with a wide range of complications being reported both during gestation and postpartum period [61, 78].

Patients with LDS are classified into 5 subtypes, based on the presence of specific mutated genes: LDS1 (TGFBR1), LDS2 (TGFBR2), LDS3 (SMAD3), LDS4 (TGFB2), and LDS5 (TGFB3). Twenty TGFBR1 and TGFBR2 are transmembrane proteins, which play a role in increased TGF-β signaling [61]. TGF-β is critical for the development and maintenance blood vessels among other functions. When overexpressed, it is thought to induce collagen and elastin abnormalities that lead to the clinical manifestations of LDS [61, 80]. Studies comparing phenotypic differences between TGFBR1 and TGFBR2 mutations have shown that aortic dissection occurs only at large diameters with TGFBR1 mutations. In addition, gender differences exist between both types: TGFBR1 mutation affected men earlier with more frequent involvement of the aorta [81].

The management of LDS involves the use of β-blockers as the first line of therapy, with or without the use of losartan, an angiotensin type II receptor blocker [80]. Due to the aggressive nature of vascular disease, however, surgical intervention of the aortic root is considered earlier as compared to MFS, potentially at any diameter above 40–45 mm [69, 82]. Many studies have cited aortic dissections taking place at diameters less than 50 mm [78]. Moreover, the high risk of aortic rupture has often necessitated aortic root replacement with prosthetic graft in LDS patients at 16.9 years. Nonetheless, recommendations for prophylactic surgery should be made on an individual basis [80]. Risks associated with the prostheses have led to the development of valve-sparing aortic root replacement (VSRR). VSRR eliminates the need for long-term anticoagulation therapy and subsequent surgeries to replace worn out prosthetic valves [83]. Recent studies on short- and mid-term outcomes of VSRR have shown superiority over traditional valve replacements in terms of survival and need for re-operation [84]. Unfortunately, there is significantly increased likelihood of pseudoaneurysms in young children following VSRR; thus, further long-term studies are needed to define the optimal surgical management strategy. In addition, ongoing surveillance imaging with CTA or MRA is warranted post operatively as well [83].

### Conclusions/Perspective for Future Work

Further studies are needed to identify key cytokines and inflammatory mediators that contribute to aortic valve damage in connective tissue disorders, which may be specifically selected for targeting in appropriate immunotherapies. This will help guide choice of therapy with anti-inflammatory agents in order to slow the progression of aortic valve damage as well as prevent the need for valve replacement. Moreover, given that many of the diseases vary in their prevalence with regard to sex, further studies are needed to investigate pathomechanisms, which may contribute to the discrepancies associated with gender.
